# Potential of targeting the alpha‐1 adrenergic receptor and phosphoglycerate kinase 1 with Terazosin to treat Alzheimer’s disease using a transgenic AD rat model

**DOI:** 10.1002/alz.089468

**Published:** 2025-01-09

**Authors:** Swastik G Pattanashetty, Lei Xie, Peter Serrano, Patricia Rockwell, Maria Figueiredo‐Pereira

**Affiliations:** ^1^ Hunter College, New York, NY USA; ^2^ The Graduate Center, City University of New York, New York, NY USA

## Abstract

**Background:**

Alzheimer’s disease (AD) is a neurodegenerative disorder without a cure. Targeting this multifactorial disease by repurposing FDA approved drugs serves as a faster mode of treatment due to its pre‐established human safety. We tested terazosin (TZ), an a‐1 adrenergic receptor (AR) antagonist and phosphoglycerate kinase‐1 (PGK1) activator as having potential to treat AD. Early stage of AD is characterized with loss of noradrenergic neurons in the locus coeruleus (LC) that initiates an over compensatory mechanism leading to increased production of norepinephrine. This causes increased synaptic activity in LC thus increasing Ab deposition via endocytosis of APP. Furthermore, dysregulated glycolysis causing decreased ATP levels is shown to play a causal role in the progression of AD and PGK1 is the first ATP generating enzyme in glycolysis.

**Hypothesis:**

TZ via its dual mechanism of action by blocking a‐1 AR and activating PGK1, will exert neuroprotective effects and ameliorate AD pathology.

**Method:**

To test this, we used the TgF‐344AD rat model that develops AD in an age dependent manner. TZ treatment starts at 5 months of age (pre pathology) until 11 months of age (moderate pathology). At 9 and 11 months of age, we used the active place avoidance test to compare the hippocampal‐dependent spatial and working memory of the transgenic not treated (TGNT) and transgenic treated (TGTR) rats with terazosin. Wild type rats were also tested as a control.

**Results:**

Our data indicate that TZ significantly improves spatial and working memory of TGTR when compared to TGNT rats. Additionally, immunohistochemical (IHC) analysis show beneficial effects of TZ in decreasing Ab plaques in cornu ammonis 1 (CA1) and subiculum (SB) subregions of hippocampus in transgenic rats. Further, ongoing analysis will reveal the effects of TZ on neuroinflammation in TGTR rats. Future studies will perform IHC on pTau and NeuN, RNAseq, western blots, and luciferase assay on the treated and untreated Tg‐AD groups to test the effects of TZ on other AD pathologies.

**Conclusion:**

Overall, this study will decipher the potential of TZ to treat AD and present new molecular candidates that can be targeted to cure or delay the progression of AD.

